# Mindfulness, cognitive fusion, and self-compassion in patients with schizophrenia spectrum disorders**—**A cross-sectional study

**DOI:** 10.3389/fpsyt.2022.959467

**Published:** 2022-08-02

**Authors:** Kerem Böge, Franziska Pollex, Niklas Bergmann, Inge Hahne, Marco Matthäus Zierhut, Selin Mavituna, Neil Thomas, Eric Hahn

**Affiliations:** ^1^Department of Psychiatry and Neurosciences, Charité – Universitätsmedizin Berlin, Campus Benjamin Franklin, Berlin, Germany; ^2^Centre for Mental Health, Swinburne University of Technology, Melbourne, VIC, Australia

**Keywords:** schizophrenia spectrum disorders, psychosis, mindfulness, self-compassion, psychological flexibility, moderated mediation, negative symptoms

## Abstract

In the last decades, third wave approaches in cognitive-behavioral therapies (CBT) have shown effectiveness in treating several mental disorders, including schizophrenia spectrum disorders (SSD). Three crucial processes associated with clinical changes in patients include mindfulness, psychological flexibility (PF) and self-compassion (SC). PF is generally assessed by cognitive fusion (CF), a negative formulated key process of PF. The current study encompasses a cross-sectional design to examine the interplay of mindfulness, CF, SC and symptom severity in SSD. It was hypothesized that mindfulness is negatively correlated with symptom severity, CF mediates the negative relation between mindfulness and symptom severity, and SC moderates the link between mindfulness and CF. In total, 79 persons with SSD were recruited at the Department of Psychiatry and Neurosciences at the Charité – Universitätsmedizin Berlin. Correlations, as well as moderated mediation analyses, were performed using the analysis modeling tool PROCESS with total symptom severity and negative symptom severity as outcome variables, measured by the Positive and Negative Syndrome Scale (PANSS) and the Self-Evaluation of Negative Symptoms Scale (SNS). Results show that the moderated mediation hypothesis was confirmed for negative symptom severity assessed by SNS, however, not for total symptom severity assessed by PANSS. In general, the association between mindfulness and CF was stronger for participants with higher SC scores in our data. Future studies should investigate the relationship between mindfulness, SC, and PF regarding symptom severity in SSD in longitudinal designs while considering the impact on different outcomes and differences regarding assessment tools.

## Introduction

Schizophrenia spectrum disorders (SSD) display a heterogenous group of complex mental disorders, encompassing negative and positive symptoms, cognitive dysfunctions (1), and affective symptoms (2), as well as comorbidities with other disorders like depression (3). Consequently, SSD is associated with high levels of distress, which influences symptom severity and can lead to chronicity affecting the overall disease burden for affected people, as well as for caregivers (3–9). The variations of distress and symptom severity among persons with SSD have been linked to coping strategies individuals use to relate to their symptoms (10–14). Therefore, changing the relationship and attitudes persons have toward themselves and their symptoms has been of rising interest in recent developments of third-wave cognitive-behavioral therapies (CBT). Throughout the past decades, third-wave CBT have been shown to be clinically effective in treating most mental disorders (15–19). Among them are acceptance and commitment therapy [ACT, (20)], compassion-focused therapy [CFT, (21)], and mindfulness-based group therapy [MBGT, (17, 22). All of them have also received increasing attention in the treatment of SSD, especially in the last decade (23). Within the framework of these psychological treatment approaches, mindfulness, self-compassion (SC), and cognitive fusion (CF) display central measurable targets. CF describes getting lost in thoughts and emotions as a negative key process influencing PF, which is the central ACT treatment mechanism (24) and is frequently used to assess PF (25). Numerous studies have shown that mindfulness, SC and components of PF are moderate to strongly correlated with each other (26–30).

The interconnectedness of these concepts is also displayed in their conceptualizations. While understood as distinct concepts, PF and SC include mindfulness as a crucial dimension (11, 31, 32). Apart from a mindful attitude toward oneself (vs. over identification with negative feelings/experiences), being self-compassionate also encompasses self-kindness (vs. self-judgment) and an understanding of one's problems as part of a common human experience (vs. feeling isolated with one's difficulties; (33). Moreover, being psychologically flexible also entails the construct of mindfulness. It refers to the ability and willingness to be fully in contact with the present moment while responding to situations in ways that facilitate valued goal pursuit (24). PF includes six interrelated dimensions that are central to ACT processes (20). Four of these, defusion, acceptance, present moment awareness, and self-as-context, are closely related to mindfulness. The other two dimensions, committed action and values, go beyond that concept and are rather behavioral change processes related to CBT guiding persons to become more engaged and active in their lives (34). PF is hypothesized to be negatively impacted by two core processes: cognitive fusion, namely getting caught in thoughts or emotions, and experiential avoidance, referring to the avoidance or repression of thoughts and emotions (35–37). PF displays a complex construct describing the mechanism of change in the ACT process. It is often assessed by the revised version of the Acceptance And Action Questionnaire [AAQ-II, (38)], which has been criticized regarding methodology and construct validity (39). Another frequently used questionnaire is the Cognitive Fusion Questionnaire [CFQ, (25)], assessing cognitive fusion as a negative key process affecting PF. Although the AAQ-II and CFQ are highly correlated, the CFQ provides better prediction in the context of different outcomes (17, 25).

Throughout the past decade, a growing body of research has started to examine the effects of mindfulness, PF, and SC in SSD. It has been shown that mindfulness, components of PF, and SC have a moderate to strong negative correlation with distress and symptom severity in SSD (7, 11, 17, 31, 32, 40, 41). A recent study found components of PF and SC to mediate the relationship between mindfulness and personal recovery of individuals with mental illnesses, including SSD (42).

In general, components of PF have been shown to mediate treatment outcomes in third-wave CBT, especially in ACT (20, 43). Additionally, ACT has also been shown to increase SC in healthy and clinical populations (44–46). At the same time, it has been found that the effects of ACT differ for different baseline levels of SC. Especially for individuals with lower baseline SC, ACT has been shown to be less effective (44). This is especially important for stigmatized identities, such as persons with SSD, who are often prone to internal shame and self-criticism and tend to have lower levels of SC (27, 44).

Overall, these findings suggest mindfulness, CF as a component of PF, and SC play an important role as mechanisms of change in third-wave CBT, which have recently been presented to be effective in the treatment of negative symptoms (19, 47, 48). Negative symptoms of schizophrenia are a crucial target, as their effective treatment remains an unmet therapeutic challenge (49). Therefore, it is crucial to investigate the relationship between the concepts of mindfulness, CF, and SC and their association with symptom severity, especially with regard to negative symptoms. Altogether, until today to the best of the authors' knowledge no study has examined all three constructs in individuals with SSD.

**Figure 1:**
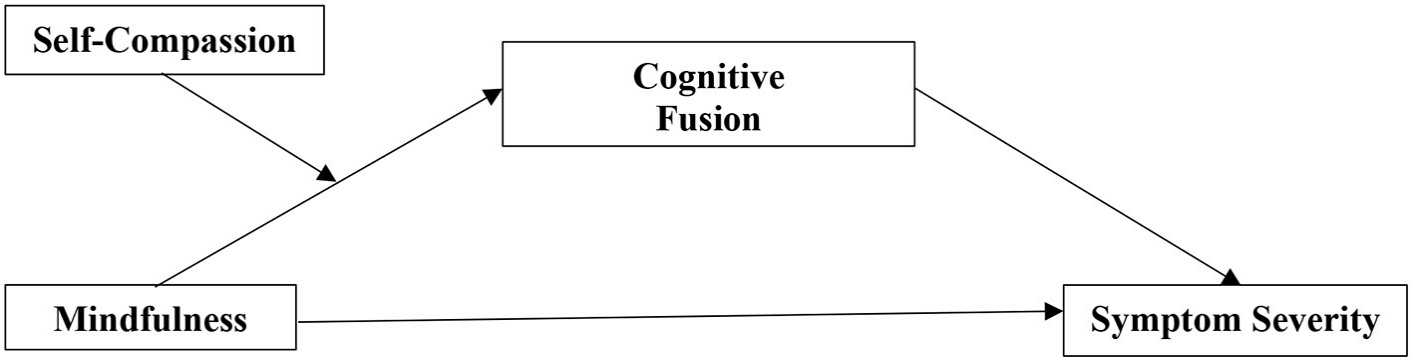
Moderated mediation model for mindfulness, self-compassion, cognitive fusion, and symptom severity.

The present study tries to fill this research gap and gain first insights into the interplay of mindfulness, CF, SC, and symptom severity to facilitate an understanding of the interplay of these dimensions in persons with SSD. A cross-sectional study design is used to disentangle how these interconnected concepts interrelate. Based on the findings of previous research, it is hypothesized that: (1) mindfulness is negatively correlated with total symptom severity and negative symptom severity, (2) CF mediates the relation between mindfulness and symptom severity, with lower CF being associated with lower symptom severity, and that (3) SC moderates the relation between mindfulness and CF, so that higher SC is associated with lower CF. The proposed model is visualized in Figure 1.

## Methods

### Design and participants

A cross-sectional design was used to achieve the main objectives of the study. Participants were recruited between March and August 2021 at the out- and inpatient facility of the Charité – Universitätsmedizin Berlin, Department of Psychiatry and Psychotherapy, Campus Benjamin Franklin, Germany. Suitable participants were identified and invited to participate in the study by a trained research assistant. Inclusion criteria were (a) current treatment at the in- or outpatient facility, (b) a diagnosis from the schizophrenia spectrum according to ICD-10 code F2x.x given by a trained psychiatrist, (c) age of 18–65 years, (d) proficiency in the German language, and (e) being able to give informed consent. Exclusion criteria were neurological diseases and severe traumatic brain injuries assessed by a trained psychiatrist.

In total, 79 participants were recruited, 14 at the inpatient facility and 65 at the outpatient facility. After giving informed consent, participants provided sociodemographic information and completed four self-rating questionnaires, followed by the rater-based Positive and Negative Syndrome Scale [PANSS; (50)]. The average duration was 65 min. All data were recorded tablet-based via REDCap software and encrypted and processed with a study-specific structure of a proprietary data management software. All data were securely and pseudonymously stored and processed. The study was approved by the Charité – Universitätsmedizin Berlin ethics committee (EA1/067/21).

### Assessments

#### Positive and negative symptoms

Positive and Negative Syndrome Scale. The Positive and Negative Syndrome Scale (PANSS) is a 30-item rater-based scale to assess the presence and severity of positive and negative symptoms, as well as general psychopathology like symptoms of depression and anxiety (50). Each item is rated by the interviewer using a seven-point format (from 1= absent to 7 = extreme) with detailed anchor descriptions. The PANSS is reported to have satisfactory internal consistency, good interrater reliability, and construct validity [α = 0.73– 0.83, (51)]. In the present study, the internal consistency of the PANSS was satisfactory (Cronbach's Alpha (CA) for PANSS total scale = 0.75, PANSS positive scale = 0.70, PANSS negative scale = 0.75, PANSS general scale = 41).

Self-Evaluation of Negative Symptoms Scale. To assess negative symptoms, a self-rating scale was additionally used. The Self-Evaluation of Negative Symptoms Scale [SNS, (52)] allows patients to evaluate themselves on five dimensions (social withdrawal, emotional range, alogia, avolition, and anhedonia) consisting of 20 items. Each item is scored on a three-point Likert scale (2 = strongly agree, 1 = somewhat agree, 0 = strongly disagree), leading to a total score ranging from 0 (no negative symptoms) to 40 (severe negative symptoms). Good psychometric properties of the SNS were replicated in multiple validation studies [α = 0.88; (53–56)]. In the present study, the internal consistency of the SNS was good (CA = 0.85).

#### Mindfulness

The Southampton Mindfulness Questionnaire [SMQ; (57) is a 16-item measure of mindful awareness of distressing thoughts, images, and perceptions. Items are rated on a seven-point fully anchored Likert scale, yielding a range of 0 to 96. The SMQ consists of four related bipolar constructs of trait mindfulness: (1) decentered awareness, (2) letting go, (3) non-judgment, and (4) non-aversion. The German version of the SMQ was used, which showed good psychometric properties and is explicitly validated for assessing mindfulness in individuals with SSD [α = 0.89, (58)]. In the present study, the internal consistency of the SMQ was good (CA = 0.83).

#### Psychological flexibility

The Cognitive Fusion Questionnaire [CFQ, (25)] assessed psychological inflexibility. The CFQ is a self-report measure consisting of seven items rated on a 7-point Likert scale (7 = always to 1 = never true), that showed high internal consistency in previous studies [α = 0.89–0.93, (25)]. In the present study, the internal consistency of the CFQ was excellent (CA = 0.91).

#### Self-compassion

The Self-Compassion Scale [SCS, (59)] is a 26-item questionnaire answered on five-point Likert scales. It measures self-compassion by separately assessing its three components, self-kindness, common humanity, and mindfulness as well as their opposites self-judgment, isolation, and over identification. In addition to the six separate facets, the combined global self-compassion score can be calculated by reversing the polarity of the negative subscales and calculating a mean throughout all subscales. The SCS has been shown to be a valid and reliable instrument regarding the total score (α = 0.91), as well as the subscales [0.66 ≤ α ≤ 0.83; (59)]. In the present study, the internal consistency of the SCS was satisfactory regarding the total score (CA = 0.73), as well as the subscales (0.66 ≤ α ≤ 0.73).

### Statistical analysis

All analyses were conducted with RStudio, version 1.3.1093. For hypotheses 1 to 3, the following statistical methods were used: First, Pearson's correlation coefficients were calculated to test whether the variables are associated with each other. Plots were visually inspected to assess linearity, normality, homoscedasticity, and potential outliers. The Rainbow and the Breusch-Pagan test were also used to test linearity and homoscedasticity. After verifying these assumptions, two moderated mediation analysis were carried out using PROCESS Model 7 (60), with total symptom severity and negative symptom severity being the dependent variables, mindfulness being the independent variable, psychological inflexibility as a mediator variable, and self-compassion as a moderator variable. Bootstrapping with 10,000 estimates was employed to compute the confidence intervals and inferential statistics. Effects were considered significant when the confidence interval did not include zero. The required sample size of a minimum of *n* = 75 was given for hypotheses 1–3, indicated after a Monte Carlo Power Analysis for indirect effects (61) to reach a power of 0.8 with a significance level of α = 0.05.

## Results

### Sample description

The mean age of the participants was 42.29 years (*SD* = 13.41, range 20 to 65). Fifty-four participants (68%) identified with the male gender, 24 as female, and 1 diverse. The participants had an SSD diagnosis for an average of 17.44 years when participating in the present study (*SD* = 12.37; range: 1–46). Sixty-nine participants indicated German citizenship, two Turkish citizenship, and eight others regarding nationality. Further sample descriptions are displayed in Table 1.

**Table 1:** Sociodemographic characteristics.

**Variables**		**Summary statistic – *n* (%)^a^**
Age (years) – mean (SD)		42.29 (13.41)
Diagnosis		
	F20 F21 F22 F23 F25	64 (81.01%) 1 (1.27%) 1 (1.27%) 2 (2.53%) 11 (13.92%)
Recruitment site		
	Outpatient Inpatient	65 (82.28%) 14 (17.72%)
Duration of disease (years) – mean (SD)		17.44 (12.37)
Gender		
	Male	54 (68.35%)
	Female	24 (30.38%)
	Diverse	1 (1.27%)
Nationality		
	German	69 (87.34%)
	Turkish	2 (2.53%)
	other	8 (10.13%)
Marital status		
	Unmarried	60 (75.95%)
	Married	7 (8.86%)
	Divorced	11 (13.92%)
	Widowed	1 (1.27%)
Housing situation		
	Private flat Shared flat Assisted living Unsettled	66 (83.54%) 1 (1.27%) 10 (12.66%) 1 (1.27%)
Children		
	Yes No	18 (24.05%) 61 (79.75%)
Educational level		
	Primary school	9 (11.39%)
	Secondary school	21 (26.58%)
	A-levels	17 (21.52%)
	Technical college	6 (7.59%)
	Apprenticeship	13 (16.46%)
	Studied	13 (16.46%)
Work situation		
	Unemployed Retired Student/apprentice Self-employed Employed Other	19 (24.05%) 20 (25.32%) 7 (8.86%) 6 (7.59%) 22 (27.85%) 5 (6.33%)

N = 79.

a Statistics in this column are n (%) unless otherwise specified.

### Zero-order correlations

As predicted in Hypothesis 1, there was a negative zero-order correlation between mindfulness assessed by the SMQ and PANSS total symptom severity (*r* = −0.33, *p* < 0.001). Although SMQ and SNS showed a non-significant zero-order correlation, mediation models were still examined for both the PANSS and SNS as measures of symptom severity, since the indirect effect is considered central to mediation models, also in the absence of a statistically significant direct effect according to Rucker et al. and Zhao et al. (62, 63).

Calculation of the zero-order correlations between the CFQ (the mediator) and these two symptom severity variables revealed positive correlations for both measures (PANSS: *r* = 0.26, *p* < 0.05; SNS: *r* = 0.33, *p* < 0.01). A negative correlation was also observed between mindfulness (the independent variable) and the CFQ (the mediator) (*r* = −0.56, *p* < 0.01). The PANSS subscales positive scale and negative scale differed in correlation patterns with the observed variables compared to the PANSS general scale. Whereas, mindfulness showed a negative correlation with the PANSS positive scale (PANSS positive scale: *r* = −0.30, p < .05), it did not show a significant correlation for PANSS negative scale. The CFQ had no significant correlation with the PANSS positive or negative scale.

The moderator SC (SCS) was significantly negatively correlated with the CFQ (*r* = −0.56, *p* < 0.01) and positively with mindfulness (*r* = 0.51, *p* < 0.01). While only the self-judgment subscale of the SCS was positively correlated with the PANSS negative scale (*r* = 0.54, *p* < 0.01), no significant correlation was found between other scales of the SCS and the PANSS. However, SCS total scale was significantly associated with the SNS (*r* = 0.54, *p* < 0.01). Exploratively, the negatively and positively formulated SCS subscales were examined. Only the negative formulated SCS subscales (self-judgment, isolation, and over-identification) were significantly correlated with the SNS (results displayed in Table 2). Recruitment site was added as a covariate based on the moderate correlations with predictors and the PANSS total score, as well as to control for the acuteness of symptoms at the moment of assessment. Zero-order correlations of the covariate recruitment site and questionnaire scores are visualized in Table 3, and descriptive statistics of questionnaire scores in Table 4.

**Table 2:** Correlations for symptom severity and SCS subscales.

**Variables**	**1**	**2**	**3**	**4**	**5**	**6**	**7**	**8**	**9**	**10**	**11**	**12**
1 PANSS total	-											
2 PANSS – positive scale	0.76^**^	-										
3 PANSS – negative scale	0.74^**^	0.26^*^	-									
4 PANSS – general scale	0.94^**^	0.66^**^	0.58^**^	-								
5 SNS total	0.38^**^	0.11	0.44^*^	0.36^**^	-							
6 SCS total	−0.08	0.09	−0.11	−0.14	−0.37^**^	-						
7 SCS – self-kindness	0.10	0.14	0.08	0.04	−0.12	0.69^**^	-					
8 SCS – self-judgment	0.19	−0.02	0.28^*^	0.19	0.39^**^	−0.66^**^	−0.19	-				
9 SCS – common humanity	0.05	0.09	0.06	−0.00	−0.07	0.55^**^	0.45^**^	0.01	-			
10 SCS – isolation	0.15	−0.02	0.18	0.18	0.48^**^	−0.74^**^	−0.26^*^	0.66^**^	−0.13	-		
11 SCS – mindfulness	−0.01	0.04	0.02	−0.06	−0.04	0.59^**^	0.61^**^	0.05	0.64^**^	−0.14	-	
12 SCS – over-identification	0.08	−0.03	0.06	0.14	0.28^*^	−0.69^**^	−0.19	0.60^**^	−0.08	0.66^**^	-0.13	-

N = 69,

* p < 0.05,

** p < 0.01.

1 PANSS, Positive and Negative Syndrome Scale; SNS, Self-Evaluation of Negative Symptoms Scale; SCS, Self-Compassion Scale.

**Table 3:** Correlations.

**Variables**	**1**	**2**	**3**	**4**	**5**	**6**	**7**	**8**	**9**
1 Recruitment site	−								
2 PANSS total	0.23^*^	−							
3 PANSS-PS	0.11	0.76^**^	−						
4 PANSS-NS	0.14	0.74^**^	0.26^*^	−					
5 PANSS-GS	0.29^**^	0.94^**^	0.66^**^	0.58^**^	−				
6 SNS total	0.00	0.38^**^	0.11	0.44^**^	0.36^**^	−			
7 SMQ total	−0.32^**^	−0.33^**^	−0.30^*^	−0.10	−0.38^**^	−0.13	−		
8 CFQ	0.24^*^	0.26^*^	0.19	0.11	0.31^**^	0.33^**^	−0.65^**^	−	
9 SCS total	−0.31^**^	−0.08	0.09	−0.11	0.14	−0.37^**^	0.51^**^	−0.56^**^	-

N = 79,

* p < 0.05,

** p < 0.01.

PANSS, Positive and Negative Syndrome Scale; SNS, Self-Evaluation of Negative Symptoms Scale; SMQ, Southampton Mindfulness Questionnaire; CFQ, Cognitive Fusion Questionnaire; SCS, Self-Compassion Scale.

**Table 4:** Descriptive statistics of questionnaire scores.

**Variables**	**Min - Max**	***M*** **(SD)**
PANSS total scale	30–210	72.86 (13.60)
PANSS – *positive scale*	7–49	16.35 (4.52)
PANSS – *negative scale*	7–49	19.78 (4.83)
PANSS – *general scale*	16–112	36.72 (7.00)
SNS total scale	0–40	13.92 (7.27)
SMQ total scale	0–96	47.78 (13.99)
CFQ	7–49	25.85 (10.45)
SCS total scale	1–5	3.12 (0.59)

N = 79, PANSS, Positive and Negative Syndrome Scale; SNS, Self-Evaluation of Negative Symptoms Scale; SMQ, Southampton Mindfulness Questionnaire; CFQ, Cognitive Fusion Questionnaire; SCS. Self-Compassion Scale.

### Moderated mediation

**Figure 2:**
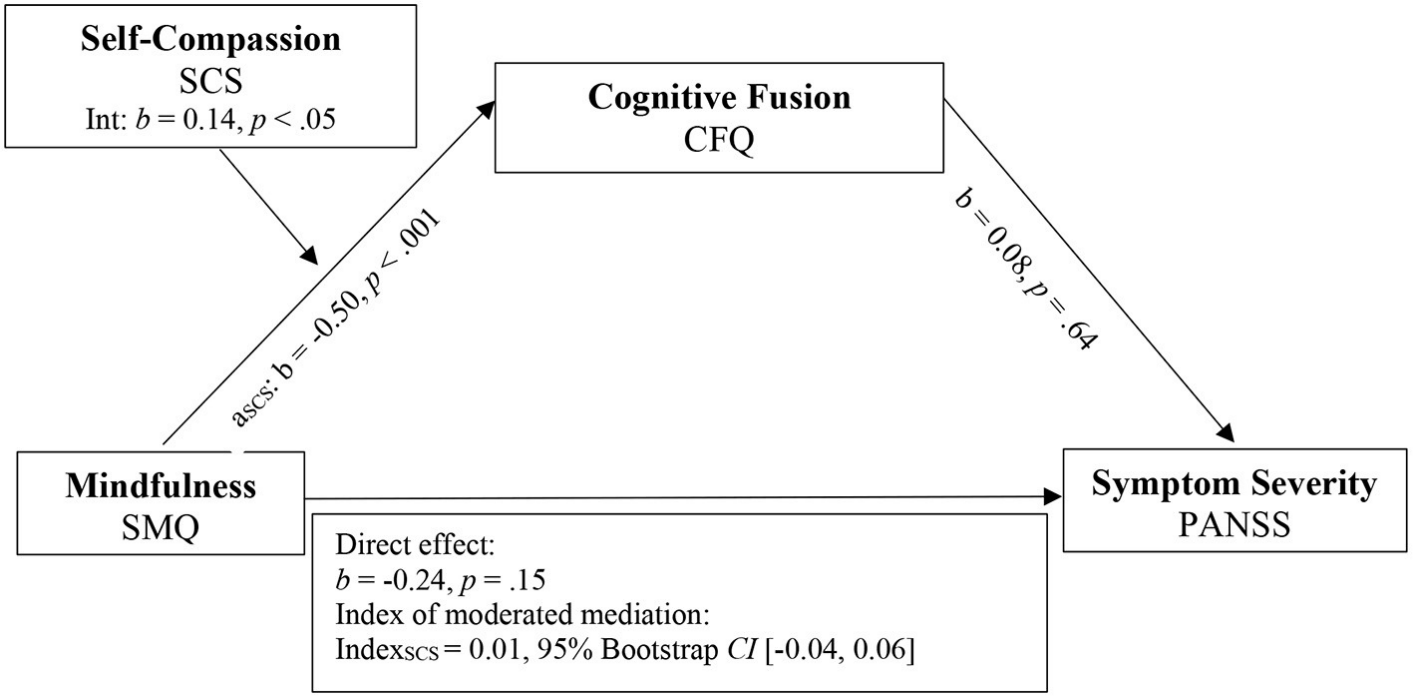
Model 1. Moderated mediation model for mindfulness, self-compassion, cognitive fusion, and symptom severity. SMQ, Southampton Mindfulness Questionnaire; CFQ, Cognitive Fusion Questionnaire; PANSS, Positive and Negative Syndrome Scale; SCS, Self-Compassion Scale.

**Figure 3:**
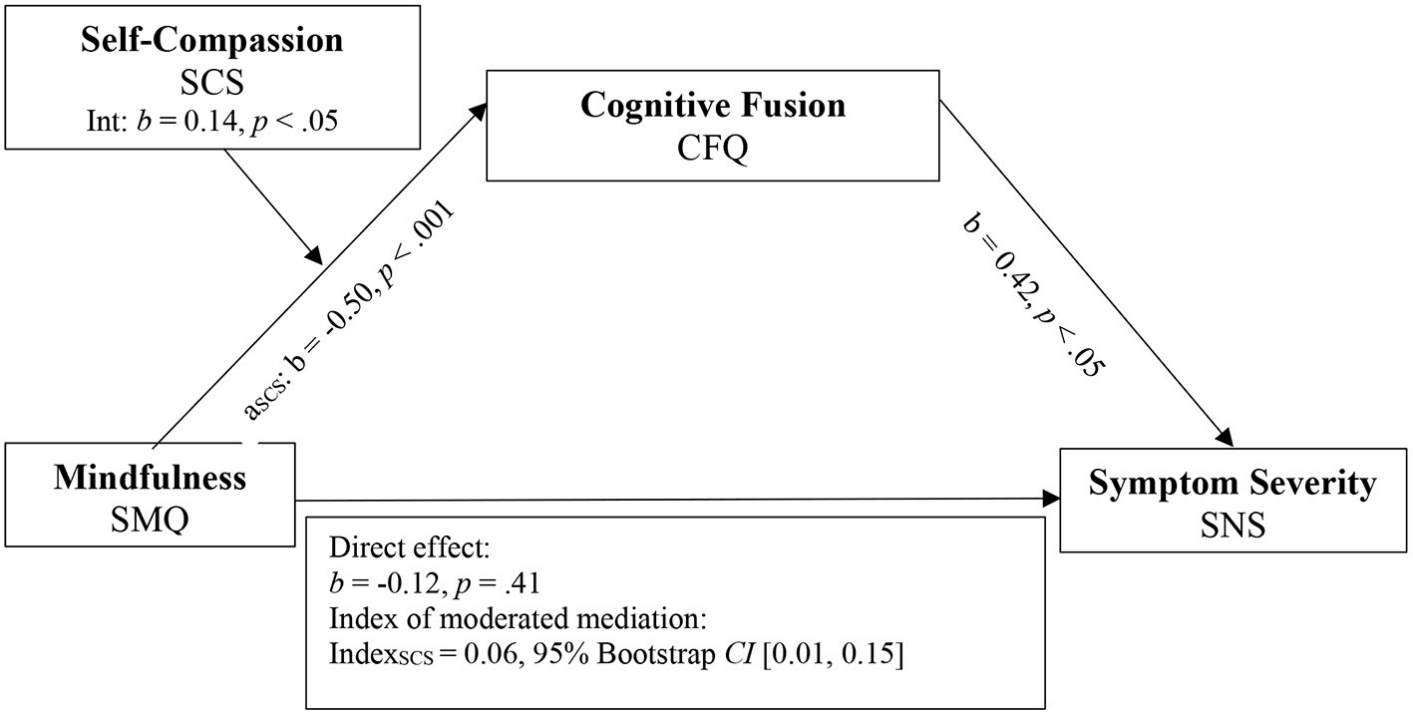
Model 2. Moderated mediation model for mindfulness, self-compassion, cognitive fusion, and negative symptom severity. SMQ, Southampton Mindfulness Questionnaire; CFQ, Cognitive Fusion.

To test the proposed model, two separate moderated mediation analyses were conducted using Model 7 of the PROCESS macro (64) with rater-based total symptom severity (PANSS) and self-rated negative symptom severity (SNS) as outcomes. Both models included the independent variable mindfulness (SMQ), the mediator variable PF (CFQ), the moderator variable SC (SCS), and recruitment site as the covariate. Results are visualized in Figure 2 for PANSS outcome measure and Figure 3 for SNS outcome measure.

The mediation analysis reached significance for SNS [*R*^2^ = 0.12, F (3, 65) = 3.49, *p* < 0.05] and PANSS as outcome measure (*R*^2^ = 0.13, F (3.75) = 3.26, *p* < 0.05). When compared to the zero-order correlations of mindfulness on total and negative symptom severity before the mediator CFQ was added, direct effects in the moderated mediation model decreased in both models (PANSS: *b*_*simple*_= −0.33, *p* < 0.1 to *b*_*model*1_= −0.24, *p* = 0.15; SNS: *b*_*simple*_= −0.13, *p* = 0.25 to *b*_*model*2_= −0.12, *p* = 0.41). When PANSS was used as outcome measure, the direct effect in the moderated mediation model was non-significant, compared to the significant zero-order correlation.

The interaction effect between mindfulness and the moderator SC was statistically significant (*b* = 0.14, *t* (78) = 2.43, *p* < 0.05), supporting the hypothesis that SC influences the negative relationship between mindfulness and CF. Detailed results of the conditional effects of mindfulness on CF for different values of SC are visualized in Table 5.

**Table 5:** Conditional effects of mindfulness at values of self-compassion.

**Moderator^a^**	**B**	**SE**	**LLCI**	**ULCI**
−1	−0.64^**^	0.11	−0.85	−0.43
0	−0.49^**^	0.10	−0.69	−0.30
1	−0.35^*^	0.12	−0.60	−0.10

* p < 0.05,

** p < 0.01,

a values of the moderator assessed by SCS for mean and one standard deviation above and below.

The indexes of moderated mediation indicated that the moderator SC significantly affected the relationship between mindfulness and CFQ for SNS, since the confidence interval did not include 0 (Index: 0.06, 95% *CI* [0.01, 0.15]). When PANSS was used as the outcome measure, however, the indirect effect of the complete moderated mediation model did not reach significance.

The covariate recruitment site did not have significant influences in any model. Detailed results about regression models and conditional indirect effects are presented in Tables 6, 7.

**Table 6:** Regression models with direct effects in the moderated mediation models for total and negative symptom severity.

**Predictor**	**Moderation model** **(DV** = **Psychological inflexibility^a^)**				
	**B**	**SE**	***t*** **(78)**	**LLCI**	**ULCI**
Constant	0.01	0.17	0.08	−0.33	0.3624
SMQ	−0.49	0.10	−4.97^**^	−0.69	−0.2962
SCS	−0.38	0.10	−3.85^**^	−0.57	−0.1817
Interaction	0.14	0.05	2.43^*^	0.03	0.2582
Recruitment site	−0.06	0.11	−0.58	−0.28	0.1529
	**Mediation model** **(DV** = **Negative symptom severity^b^)**				
Constant	0.11	0.26	0.42	−0.41	0.6323
SMQ	0.12	0.15	0.84	−0.17	0.4173
CFQ	0.42	0.16	2.60^*^	0.10	0.7483
Recruitment site	−0.08	0.20	−0.42	−0.47	0.3097
	**Mediation Model** **(DV** = **Total Symptom Severity^c^)**				
Constant	−0.24	0.26	−0.93	−0.77	0.2810
SMQ	−0.23	0.16	−1.45	−0.56	0.0871
CFQ	0.08	0.16	0.47	−0.25	0.4019
Recruitment site	0.18	0.20	0.91	−0.21	0.5722

* p < 0.05,

** p < 0.01,

a assessed by CFQ,

b assessed by SNS,

c assessed by PANSS total scale.

**Table 7:** Regression results for conditional indirect effects of mindfulness on symptom severity.

**Moderator^a^**	**Total symptom severity^b^**			
	**B**	**BootSE**	**BootLLCI**	**BootULCI**
−1	−0.05	0.10	−0.23	0.17
0	−0.04	0.08	−0.81	0.14
1	−0.03	0.06	−0.14	0.10
**Moderator** ^a^	**Negative symptom severity^c^**			
−1	−0.27	0.10	−0.47	−0.08
0	−0.21	0.80	−0.37	−0.06
1	−0.15	0.08	−0.30	−0.01

a values of the moderator assessed by SCS for mean and one standard deviation above and below,

b assessed by SNS,

c assessed by PANSS.

## Discussion

The aim of the present study was to investigate the interplay of mindfulness, CF as a component of PF, and SC in relation to symptom severity in patients with SSD. First, it was hypothesized that mindfulness and symptom severity are negatively associated. The second hypothesis investigated in this study was that CF mediates the relationship between mindfulness and symptom severity. And finally, it was hypothesized that SC would moderate the relationship between mindfulness and CF.

In line with our first hypothesis, mindfulness was significantly correlated with total symptom severity as measured by PANSS. When investigating the PANSS subscales, mindfulness was significantly correlated to positive and general symptom severity but was not significantly associated with the negative syndrome subscale of the PANSS. Similarly, mindfulness was not significantly associated with negative symptom severity as measured by SNS. However, while non-significant, the data suggest a negative correlation between mindfulness and negative symptom measures, nevertheless needed further research with larger samples. Both CF and SC had a significant relationship with negative symptom severity, as measured by the SNS, but they were not significantly associated with the negative syndrome subscale of the PANSS. Only the self-judgment subscale of the SCS was significantly associated with PANSS-NS. In contrast, previous studies investigating the relationship between symptom severity and SC in SSD found SC to be negatively correlated with positive symptoms and cognitive disorganization subscales of the PANSS (32, 66). Only when using the Narrative Compassion Interview, which uses a semi-structured interview format and systematic coding system to assess self-compassion, Gumley and Macbeth (66) found SC to be negatively related to negative symptoms. Therefore, it may be useful for future studies investigating the relationship between SC, CF, and mindfulness to incorporate self-rating and rater-based SC and symptom severity measures in larger samples.

For our second hypothesis, we found CF to significantly mediate the relationship between mindfulness and the subjective experience of negative symptom severity, as measured by the SNS. This is in line with previous research suggesting CF as a core process of PF constitutes a mechanism of change in third-wave CBT in individuals with schizophrenia (20, 43). However, while research has shown components of PF to be predictors of social functioning (35), as well as individual difference factors for psychosis (37, 67), in the present study CF was not found to significantly mediate the relationship between mindfulness and overall symptom severity as measured by the PANSS. Therefore, this study highlights the central role of mindfulness and CF as a compoment of PF in treating negative symptoms in particular. However, more research is needed to understand the inconsistent findings regarding the relationship of different components and assessments of PF and mindfulness with positive symptoms.

Finally, the third hypothesis regarding the moderating role of SC on the relationship between mindfulness and CF was confirmed in both models. It was found that participants with higher mindfulness scores tended to be more psychologically flexible/showed less cognitive fusion and this effect was stronger for participants with higher SC. Especially the negatively formulated subscales of the SCS (self-judgment, over identification, and isolation) were significantly correlated with negative symptom severity. This is in line with research suggesting the value of SC practices alongside mindfulness in third-wave CBT in phases of psychosis characterized by self-stigmatization and social marginalization, which often affect negative symptoms. Not only in early psychosis, in which self-stigmatization often emerges but also in established psychosis SC has been shown to reduce social marginalization and self-stigma as well as fostering positive senses of self (68). A recent systematic review has consistently confirmed SC as a decisive factor in reducing symptom severity and improving affective symptoms in treating SSD (47).

Similarly, from a theoretical perspective, it has been suggested that increasing mindfulness may increase awareness of distressing internal events and threatening psychotic experiences; therefore, cultivating SC and acceptance may be important in diminishing these changes (68, 69). This is in line with research finding self-compassion to mediate the relationship between mindfulness and symptom severity, as well as to be both a predictor of treatment effects and a mechanism of change in mindfulness-based interventions (70–72). This study adds to understanding the effects of SC for SSD; specifically, it is suggested that fostering SC might allow individuals with SSD to reduce CF and improve PF more effectively, thereby reducing negative symptom severity. However, further research, especially randomized controlled trials, is needed to show the longitudinal effects of SC and CF/PF in treating SSD. Additionally, it would be useful to examine the role of stigma and self-criticism in third-wave CBT targeting SC and CF/PF.

Overall, this study displays the important roles of SC and CF in the relationship between mindfulness and negative symptom severity in SSD. However, while PF and SC partly intersect in their conceptualizations (29), it is important to investigate which specific aspects of SC or PF influence distress and symptom severity in SSD. In this study, only the CFQ was assessed as a negative core process of PF. In future studies, besides considering the AAQ-II, other aspects of PF should be considered more directly. From a theoretical perspective, mindfulness, CF, and SC might also overlap in other aspects, such as cognitive insight and metacognitive capacity, that are part of third wave CBTs, specifically for meta cognitive therapy (MCT) for SSD as well (41). Recent studies found correlations between metacognition and cognitive fusion (73) and increased metacognitive awareness as a predictor of greater SC (70). Another study found self-compassion as a mediator between metacognition and meaning in life (74). The interplay of CBT related constructs, as well as the degree of overlap and additional explanation of variance for SSD should be investigated in future studies. Furthermore, PF in its core meaning describes interactions between a person, their emotions, and actions, wherefore randomized controlled trials are needed to gain the necessary insights into the dynamic influence of PF in relation to mindfulness, SC, and symptom severity clearly, as well as to draw causal conclusions (75). Additionally, the usefulness of understanding the effects of mindfulness, SC, and PF at different stages of psychosis has been recently highlighted (68). Therefore, future studies should investigate how these concepts interact in different stages of psychosis.

It is noteworthy that the moderated mediation model could not be confirmed for the outcome total symptom severity as measured by the PANSS, although mindfulness was significantly associated with the PANSS total, as well as the positive and general symptoms subscales of the PANSS. Additionally, non-significant negative relationships were found between mindfulness and PANSS-NS, as well as between mindfulness and SNS. Nevertheless, the moderated mediation model using SNS as an outcome measure was confirmed. This difference in outcome can be ascribed to the distinct operationalizations of the PANSS and the SNS and the fact that the SNS is a self-rated measure, whereas the PANSS is a rater-based assessment tool. More specifically, SNS and PANSS operationalize negative symptomology differently. The PANSS-NS includes aspects not typically considered part of negative symptomologies, such as evaluations regarding cognitive functioning, depressive symptoms, inadequate behavior, and disorganized speech. Additionally, PANSS-NS does not address the concept of amotivation, which is considered in SNS (1, 65, 76). Therefore, the influence of different conceptualizations and nuances of each assessment tool should be considered in future research. Additionally, future studies should investigate whether higher SC and PF mainly reduce negative symptomology and to what degree this is influenced by the operationalization of instruments.

Lastly, the variable recruitment site served as an additional implicit measure of how acute symptoms and distress were. In our data, in-patient participants were more likely to have more severe symptoms, were less mindful, and less self-compassionate. However, in the moderated mediation models, this covariate did not have a significant influence. Only 17.9% of the participants from our sample were inpatients and this number might be too small to detect effects. Further studies should consider comparing a more balanced sample regarding acuteness of symptoms in participants with SSD.

### Limitations

Given the panel study design, it is not possible to draw causal conclusions since the temporal precedence of cause and effect is not given using correlational data. Therefore, the positions of variables within the discussed models remain interchangeable until further investigation and conclusions about the direction of the association cannot be drawn. At the initial stages of research, however, correlational data can indicate mechanisms of action, guiding future randomized controlled intervention studies (77). Although the present sample size met the required statistical minimum, it might be too small to comprise the diversity in clinical pictures displayed in SSD. Future research might include a balanced sample of in- and outpatients as well as heterogeneous samples of patients with SSD. Furthermore, examining the level of understanding of mindfulness and already existing experiences with mindfulness practices of participants would be useful in future research. To control for the level of understanding of the constructs itself might have allowed to differentiate low mindfulness scores due to actual low mindfulness skills from low scores that are due to difficulties in grasping the concept of mindfulness.

## Conclusion

This study provides new insights into the relationship between mindfulness, SC, and CF to symptom severity in SSD. CF mediated the relationship between mindfulness and self-rated negative symptom severity. Higher levels of SC strengthened the negative relation between mindfulness and CF in our sample in general. CF being a negative formulated key process of PF, this emphasizes the importance of additional aspects of PF and SC, which are behavioral change processes directed toward values and life goals, as well as the importance of considering kindness toward oneself and embedding own suffering as a part of shared and common humanity. PF should be fostered more in SSD, with the direct consideration of the prerequisite of strengthening SC. Due to the cross-sectional design of this study, no causal conclusions can be drawn until further investigation. Future studies should investigate the interplay of mindfulness, SC, and PF in longitudinal studies, considering variations in the conceptualization of negative symptoms due to assessment tools.

## Data availability statement

The raw data supporting the conclusions of this article will be made available by the authors, without undue reservation.

## Ethics statement

The studies involving human participants were reviewed and approved by Charité Universitätsmedizin Berlin. The patients/participants provided their written informed consent to participate in this study.

## Author contributions

KB designed the study, executed the study, and wrote the paper. FP designed the study, executed the study, conducted the data analyses, and wrote the paper. IH and NB collaborated with the design, execution of the study, and editing of the manuscript. MZ assisted with the editing of the final manuscript and recruitment process. SM and NT assisted with the editing of the final manuscript. EH collaborated with the design and editing of the final manuscript. All authors contributed to the article and approved the submitted version.

## Conflict of interest

The authors declare that the research was conducted in the absence of any commercial or financial relationships that could be construed as a potential conflict of interest.

## Publisher's note

All claims expressed in this article are solely those of the authors and do not necessarily represent those of their affiliated organizations, or those of the publisher, the editors and the reviewers. Any product that may be evaluated in this article, or claim that may be made by its manufacturer, is not guaranteed or endorsed by the publisher.
